# Antibacterial Properties of Dental Luting Agents: Potential to Hinder the Development of Secondary Caries

**DOI:** 10.1155/2012/529495

**Published:** 2012-03-14

**Authors:** Erik Unosson, Yanling Cai, Xiyuan Jiang, Jesper Lööf, Ken Welch, Håkan Engqvist

**Affiliations:** ^1^Division of Applied Materials Science, Department of Engineering Sciences, The Ångström Laboratory, Uppsala University, Box 534, 751 21 Uppsala, Sweden; ^2^Division of Nanotechnology and Functional Materials, Department of Engineering Sciences, The Ångström Laboratory, Uppsala University, Box 534, 751 21 Uppsala, Sweden; ^3^Doxa AB, Axel Johanssons Gata 4-6, 754 51 Uppsala, Sweden

## Abstract

A modified direct contact test was used to evaluate the antibacterial properties of four commercially available dental luting agents (RelyX Unicem, Ketac Cem, Ceramir Crown & Bridge and Harvard Cement) and two reference materials (glass-ionomer cement and calcium aluminate cement) compared to a negative-control material (PMMA). *Streptococcus mutans* bacteria were placed in direct contact with specimens that had been aged for 10 min, 1 day, and 7 days, in order to test the antibacterial properties of the materials. A metabolic assay containing resazurin was used to quantify the amount of viable bacteria remaining after the direct contact tests. The effects of pH and fluoride on bacteria proliferation were also evaluated. Strongest antibacterial properties were found for calcium aluminate cement, followed by Ceramir Crown & Bridge and RelyX Unicem. Ketac Cem, Harvard Cement, and the reference glass-ionomer cement showed bacteria content either higher than or not significantly different from the PMMA control in all instances. pH levels below 6.3 and above 9.0 were found to have negative effects on bacterial proliferation. No correlation between either acidic materials or fluoride release and antibacterial properties could be seen; rather, basic materials showed stronger antibacterial properties.

## 1. Introduction

Prosthetic dentistry revolves around the restoration or replacement of lost or missing teeth using crowns, bridges, and other dentures. In fixed partial dentures (FPDs), dental luting agents serve as the link between the prepared, supporting tooth, and the prosthetic tooth material. An ideal dental cement to be used as a luting agent should be biocompatible, inhibit caries or plaque formation, have low solubility, have correct film thickness and viscosity, have long working time and short setting time, have high strength and stiffness comparable to dentin, show no microleakage, permit easy removal of excess material, and exhibit high retention [1]. A wide range of luting agents with varying chemistries and properties are available on the market and strive to meet these high requirements. However, FPDs are prone to failure, with the number one cause of failure being secondary dental caries [2, 3]. Secondary caries is readily formed in restoration margins or gaps where plaque, a biofilm containing various bacteria, can adhere [4]. Among the bacterial species present in these biofilms, *Streptococcus mutans* is recognized as the one most frequently involved in caries formation [5]. These bacteria produce acids when metabolizing fermentable carbohydrates, which can then dissolve the calcium phosphate mineral content in enamel and dentin, eventually leading to a cavity or failure of the FPD [6]. Colonization of bacteria often occurs at secluded locations in shortage of oxygen and mechanical disturbance and consequently bactericidal properties of dental cements are of particular importance since it is one of the few means by which bacteria can be inhibited in these sites.

 Antibacterial properties of dental cements have been evaluated in the past [7–9], and the bactericidal effects are often attributed to their low pH and/or release of fluoride ions. There is, however, conflicting data as to whether the low-level fluoride release or acidity of currently used materials is sufficient for long-term bactericidal effect, and which class of cement performs the best [7, 9–11].

Two popular assays used to evaluate antibacterial properties of dental cements are the agar diffusion test (ADT) and direct contact test (DCT). While the ADT has been used successfully [7, 12], it has certain limitations. Results are semiquantitative and depend on solubility and diffusion properties of both the material tested and the medium used. To avoid the limitations of ADT, DCT was developed by Weiss et al. [13] and further used in several other studies of similar character [8, 9, 14, 15]. In DCT, outgrowth of bacteria after close contact with a nonsoluble material is quantified by continuous measurements of optical density (OD), which is proportional to the concentration of bacteria present in solution.

In this study, a modified DCT was used to evaluate the antibacterial properties of four commercially available dental cements (three acid-base reacting and one self-etching) and two acid-base reacting reference materials. A metabolic assay containing resazurin was used to quantify the bacteria present in solution after direct contact with the cements. Resazurin is a common metabolic activity indicator that has been shown to be effective in assessing bacterial viability [16] and in biofilm quantification [17]. It is a water-soluble dye that can be reduced to highly fluorescent resorufin by metabolically active bacteria. In addition, the effects of pH and fluoride on bacteria proliferation were evaluated by means of OD measurements.

## 2. Materials and Methods

### 2.1. Sample Preparation

Compositions, powder to liquid (P/L) ratio and suppliers of the dental cements investigated in this study are shown in Table 1. Commercially available products were all prepared in accordance with the manufacturers instructions. RelyX Unicem (RelyX) and Ketac Cem (Ketac) were prepared using the Aplicap Activator and Applier and mixed using a CapMix universal mixing unit (3 M ESPE). Harvard Cement (zinc phosphate, ZP), Ceramir Crown & Bridge (Ceramir), calcium aluminate cement (CA), and glass-ionomer cement (GIC) were all mixed by hand using a stainless steel spatula on a clean surface. After preparation, the cements were transferred to rubber molds, 1.5 mm deep and 5 mm in diameter. Light curing was used for RelyX, while the remaining cements were allowed to set for 7–10 min at 37°C in 100% relative humidity. After setting, samples were removed from the molds and returned to the oven for aging at 37°C in 100% relative humidity for 10 min, 1 day, or 7 days before testing for antibacterial effect. As a control, samples of poly(methyl methacrylate) (PMMA), a biomaterial considered inert in its fully polymerized state, were cut in the same dimensions from a solid rod.

### 2.2. Bacterial Strain and Growth Conditions

*Streptococcus mutans* (strain NCTC 10449) was used to determine growth inhibition activity of the investigated cements, the effects of varying pH, and the bactericidal effect of fluoride. *S. mutans* has frequently been used to test antimicrobial activity [12, 18–21] and is considered a primary etiological agent of caries [5]. *S. mutans* was inoculated in brain-heart infusion (BHI) broth (Sigma-Aldrich, Steinheim, Germany) and cultured anaerobically at 37°C to exponential phase (OD_600_ = 1.0). The culture was centrifuged at 4000 rpm for 5 min and the bacteria pellet was resuspended in sterile H_2_O. The concentration of bacteria was adjusted to OD_600_ = 1.0, which corresponds to 10^9^ cfu/mL.

### 2.3. Direct Contact Test

The direct contact test (DCT) performed by Weiss et al. [13] is based on turbidimetric determination of continuous bacterial outgrowth from the material under investigation. In the modified direct contact test performed in the current study, resazurin was used as metabolic activity indicator to quantify viable bacteria instead of OD measurements. In the resazurin assay, blue, nonfluorescent resazurin is reduced by metabolic intermediates (e.g., NAPDH) to pink resorufin, which is fluorescent and therefore a sensitive indicator of the amount of viable bacteria in the assay [16].

Six samples of each cement type and a control group of six PMMA samples were tested for each aging time. Each sample was placed at the bottom of a well of a 96-well plate (sterile, transparent, flat bottom, CELLSTAR, Greiner Bio-One GmbH, Germany) and 5 *μ*L of the *S. mutans* suspension was added and evenly distributed on the sample surface. Samples were then incubated at 37°C for 1 h, during which time the suspension liquid evaporated, ensuring direct contact between bacteria and testing material. Immediately following this step, 135 *μ*L of Mueller Hinton (MH) broth culture medium (Fluka) and 15 *μ*L resazurin (1.25 *μ*g/mL) were added to each well and incubation was continued at 37°C for 100 min, after which fluorescence measurements of each well were made using a multimode microplate reader (Infinite 200 PRO, TECAN, Männedorf, Switzerland) set to 530 nm excitation and 590 nm emission wavelengths [22]. Samples were removed from the wells prior to fluorescence measurement since the testing materials themselves may influence the measurements.

In order to calibrate results from the sample viability assays, a standard curve was made from a set of resazurin assays containing a serial dilution of bacteria. The standard curve provides a quantitative measure of viable bacteria in the sample assays by comparing the fluorescent signals from the sample assays to that of the serial dilution.

### 2.4. Antibacterial Effects of pH

For measuring the effects of varying pH on bacterial proliferation, 10 different buffer solutions (Sigma-Aldrich) ranging from pH 1 to 11 were used. Test solutions comprised of a suspension of *S. mutans* in BHI broth and pH buffer were prepared as detailed in Table 2. A solution containing sterile deionized H_2_O (hereafter referred to as H_2_O) instead of pH buffer was used as control. The pH of H_2_O is around 7 and is not expected to have negative effect on bacterial growth. The resulting pH of the control and test solutions was measured using a pH meter (HI 83141, Hanna instruments). Solutions were then incubated at 37°C and OD measurements at 600 nm were made on 3 mL aliquots from each solution using a spectrophotometer (UV-1650, Shimadzu, Kyoto, Japan) after 0 to 10 hours of incubation. For these tests, OD measurements were used because it offered a simple and reliable means of measuring the concentration of bacteria as a function of time. Three different aliquots from each solution were measured at each time, after which they were returned to their respective solutions. A reference solution containing only BHI broth culture medium and H_2_O was used to calibrate the instrument at 600 nm and create a baseline.

### 2.5. Antibacterial Effects of Fluoride

Sodium fluoride (NaF) powder (Sigma-Aldrich), H_2_O, and BHI broth were mixed to obtain 10 mL solutions with varying fluoride strength, from 0 to 2000 ppm. To each solution, 50 *μ*L of *S. mutans* suspension (OD_600_ = 1.0) was added. Three samples at each fluoride concentration were incubated at 37°C for 8 h, after which OD measurements were made at 600 nm in the spectrophotometer.

### 2.6. Statistical Analysis

Using the statistical software package SPSS v19 (SPSS Inc., IL, USA), a univariate analysis of variance (ANOVA) was carried out to identify statistical differences in amount of viable cells after DCT on the different materials (cements and control) and after varying time. Multiple comparisons were made using the Sheffe's method, and differences were considered significant at the 95% confidence level (*P* < 0.05). 

## 3. Results 

### 3.1. Direct Contact Test

**Figure 1:**
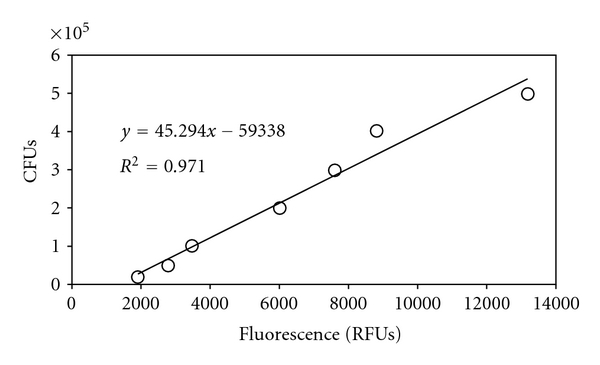
Relationship between CFUs and fluorescence, derived from the standard curve of serially diluted bacteria.

**Figure 2:**
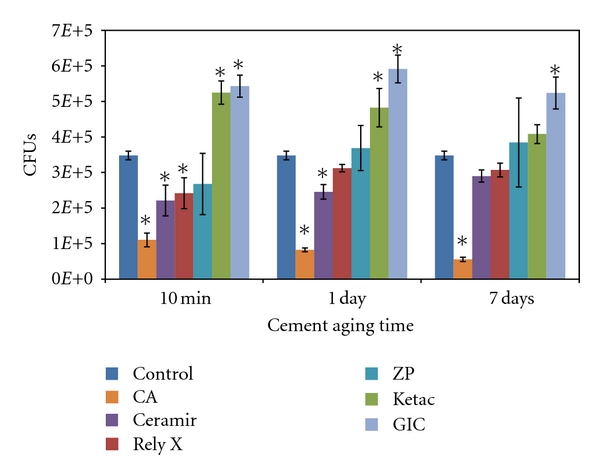
Amount of CFUs present in solution after direct contact tests. Significant differences (*P* < 0.05) from the control (PMMA) are indicated by *. Note: The first bar in each aging time group shows the same control PMMA data for each group since the PMMA samples were not aged.

The standard curve from the dilution series resulted in a linear relationship between the degree of fluorescence and the known amount of bacteria present.  Figure 1 shows the corresponding relationship between the number of viable bacteria, labeled as colony forming units (CFUs) and fluorescence, measured in relative fluorescence units (RFUs). A linear fit to the data was used to calibrate fluorescence measurements from the test cements relative to the dilution series and was utilized in the results shown in Figure 2.

Figure 2 displays the number of viable bacteria for each material after the different aging times, where each bar is the average from six different wells. The strongest antibacterial activity was demonstrated by CA, which showed significantly different values from the control (*P* < 0.0005) and all other cements (*P* ≤ 0.014) at all aging times. It also showed a significant decrease in CFUs with increased aging time (*P* ≤ 0.006). Ceramir showed antibacterial properties after 10 min and 1 day of aging (*P* ≤ 0.004) but was not significantly different from the control after 7 days of aging. RelyX also showed significant difference from the control after 10 min aging (*P* = 0.022), but not after 1 day or 7 days. The remaining cements (ZP, Ketac and GIC) showed either higher number of viable bacteria or were not significantly different from the PMMA control at all instances. 

### 3.2. Antibacterial Effects of pH

**Figure 3:**
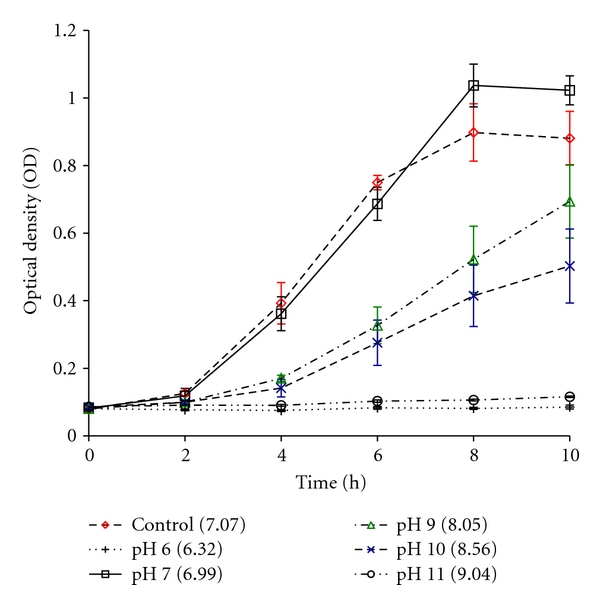
Growth curves of *S. mutans* in BHI at different pH buffer/BHI solutions. Value in brackets indicates the resulting pH of buffer/BHI solution.

**Figure 4:**
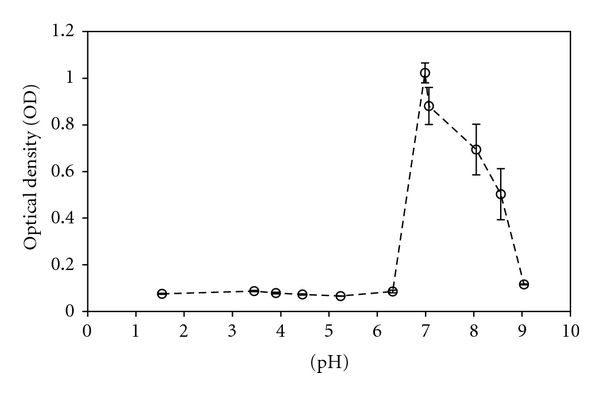
Amount of bacteria present in solution after 10 h incubation as a function of solution pH. Proliferation was seen at neutral and slightly basic levels.

Effects of pH on bacterial proliferation are shown in Figures 3 and 4. The pH of the control and test solutions ranged from 1.5 to 9.0. No bacterial proliferation was seen at levels below pH 6.3 or above pH 9.0. *S. mutans* proliferated between pH 6.3 and 8.6 and showed strongest growth in the neutral pH buffer and in the H_2_O control. The curves are characterized by an initial lag phase (0–2 h), followed by a growth phase and a final stationary phase. However, a considerable gap exists between the pH buffer 7 and H_2_O control in the latter part of the growth. This is likely due to the fact that as bacteria grow, acid is produced that will negatively affect the bacterial proliferation after approximately 6 h. The buffer, however, has the ability to regulate for this and proliferation continues until the stationary phase is reached at a later point. 

### 3.3. Antibacterial Effects of Fluoride

**Figure 5:**
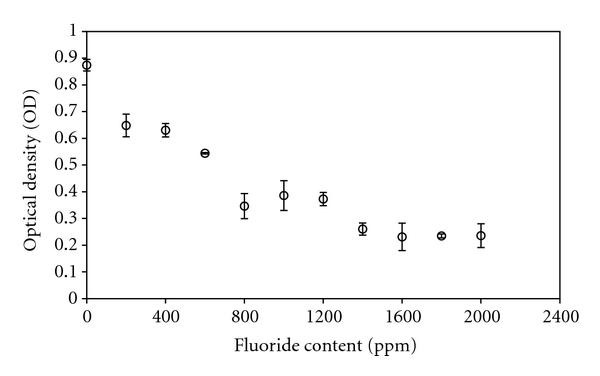
Amount of *S. mutans* present in solution as a function of fluoride content, measured by OD at 600 nm, after 8 hours incubation.

Figure 5 displays the effect of fluoride on bacterial proliferation. A negative effect (i.e., reduction in the bacterial growth rate) was observed even at 200 ppm, with an increasing effect with increasing fluoride concentration until an approximately steady state was reached at roughly 1400 ppm. 

## 4. Discussion 

The longevity of dental restorations is often determined by their ability to resist plaque formation and consequently avoid secondary caries [2, 3, 20]. Although it has been suggested that early plaque formation is largely influenced by the intraoral positioning of restorations and general oral environment [4], cements having antibacterial properties could successfully prevent or delay caries formation and prolong the lifetime of restorations. In the current study, antibacterial properties of different classes of dental cements were investigated by means of a modified DCT. Previous studies have shown various glass-ionomer cements to have initial bactericidal properties due to low pH during curing or slow release of fluoride [7, 8, 10, 23], whereas another study showed polycarboxylate cement and zinc phosphate cement to be more active both initially and over time [9], which was attributed to low pH and fluoride release. In this study, the bioceramics CA and Ceramir exhibited the highest degree of antibacterial activity, followed by the RelyX resin cement. ZP showed some initial bacterial inhibition but was not significantly different from the PMMA control and displayed a large spread in data. The pure GIC and Ketac showed higher bacterial content than the PMMA control for all aging times, and the differences were significant in all cases except for Ketac after 7 days of aging. 

 Conventional luting cements that set by acid-base interactions produce an environment that is initially acidic, but approach neutrality during the course of the reaction. Low initial pH is recognized as beneficial with regards to bactericidal properties; however, this can have negative consequences as acid diffusion through thin dentin sections which can cause pulpal irritation has been reported for glass-ionomer and zinc phosphate cements [24]. The minimum pH for organisms such as *S. mutans* to grow is approximately 5 [25], and although this study showed clear inhibition from pH 6.3, none of the cements maintaining a pH below neutral after setting (Ketac, GIC, and ZP all end up around pH 6) displayed any clear bactericidal properties. RelyX showed some antibacterial properties after being aged for 10 min, but at that time should no longer be acidic. The light cured RelyX resin cement reaches pH 5 within minutes after onset of the reaction and quickly continues to pH 8 before stabilizing at a neutral level after 24 h [26]. This means that the initial bactericidal effect of RelyX could potentially be attributed to fluoride release which is highest during the first 24 h [10]. However, GIC also releases fluoride and here no antibacterial effect was observed at any aging time, so it is not clear that fluoride gives an antibacterial effect. 

Ceramir is also acidic during the first stages of the setting reaction, but quickly surpasses neutrality to reach basic levels. The pH of CA can reach over 12 during the reaction [27], but settles around 11.5 after final setting, while Ceramir stabilizes around 8.5 after roughly 12 hours. As shown in Figure 4, inhibition was also observed for higher levels of pH, and therefore the bactericidal properties displayed by Ceramir during initial setting times and especially by CA could be due to the basic environment they produce. Since the pH of Ceramir lowers after 12 h, it is also likely that another antibacterial mechanism, such as fluoride release, is responsible for the observed antibacterial effect. 

 Fluoride is not only widely used as an anticariogenic agent in common dental products such as toothpaste and mouthwash, it is also present in a wide range of restorative materials [10]. Among the cements investigated in the current study, fluoride is released from RelyX, Ceramir, Ketac, and GIC. However, within the limitations of the experiments performed, no conclusive evidence of antibacterial activity directly attributable to fluoride release from these cements could be shown. The anticariogenic effects of fluoride involve a variety of mechanisms, including inhibition of demineralization and enhancement of remineralization at the crystal surface as well as inhibition of bacterial growth and metabolism [6]. This discussion will, however, be limited to the action of fluoride on bacteria. 

As seen in Figure 5, fluoride has a direct effect on bacteria proliferation. The decreasing OD with increasing fluoride concentration indicates that increasing fluoride concentration negatively affects the bacterial growth rate. Fluoride can act in various ways to stop or slow bacterial growth; F^−^ or HF can cause direct inhibition of enzymes such as enolase, urease, phosphatases, or heme catalase, which are all involved in microbial cell metabolism; metallic fluoride complexes such as AlF^−^ and BeF^−^·H_2_O can effect enzymes and regulatory phosphates, with either positive or negative outcome for the cell; and HF can act as a transmembrane proton carrier to disturb the cell membrane through a ΔpH discharge [25]. Direct inhibition of enzymes through binding of F^−^/HF is a pH-dependent process, where F^−^ in many cases binds to sites normally occupied by OH^−^, followed by binding to a proton. F^−^ has been shown to be unable to cross the cell wall and membrane, whereas HF passes unhindered [25]. Well inside the cell, HF dissociates to create an acid environment and releases F^−^, which will accumulate and disturb the enzymes present [6]. In this case, HF acts as a proton carrier across the membrane and overloads the proton-extruding ATPases, as extruded protons will reenter as HF and eventually cause cell starvation and generally deenergize the membrane through the ΔpH discharge. 

 It has been suggested that the ideal release profile of fluoride from dental cements should be characterized by an initial burst, followed by a stable, lower release rate [28]. It should be noted that the amount of fluoride released from dental restoratives is far less than the amounts shown in Figure 5. Accumulated release varies between material types and products, but resin cements have been shown to have similar release profiles as resin-modified and conventional glass-ionomer cements, which are able to reach 15 ppm in the first week [20, 28, 29]. However, with the occurrence of shrinkage during setting, which allows gaps or voids to form between the cement and tooth, a strong initial fluoride release could increase fluoride concentration in the area, reducing the proliferation of bacteria and helping prevent secondary caries. A subsequent constant, lower level release of fluoride could aid in maintaining the resistance to bacteria and buildup of plaque and subsequent caries in sensitive areas. 

Based on the results from the DCT in the current study, fluoride released from the cements could not be singled out as an antibacterial agent. Similar conclusions have been drawn for fluoride releasing restoratives by others [10, 20]. Nonetheless, it is possible that synergetic effects of fluoride with, for example, pH result in the observed bactericidal effects with luting agents such as Ceramir.

## 5. Conclusions 

The antibacterial properties of four commercially available luting agents and two reference materials were tested in this study. Only calcium aluminate cement showed significant antibacterial properties compared to the PMMA control after aging for 10 min, 1 day, and 7 days. An increase in antibacterial effect was found for calcium aluminate cement with increased aging time. Antibacterial activity was also shown for Ceramir after 10 min and 1 day aging and for RelyX after 10 min aging. Cements having high rather than low pH after setting were shown to be more antibacterial, despite the fact that clear negative effects on bacteria proliferation were seen at pH levels below 6.3 and above 9.0. No correlation between either acidic materials or fluoride release and antibacterial properties could be seen; rather, basic materials showed the strongest antibacterial properties. 

## Figures and Tables

**Table 1:** Contents, type, supplier, P/L ratio, and possibility of fluoride release of the dental cements investigated.

	Powder	Liquid	Type	Supplier	P/L ratio	Fluoride release
RelyX Unicem	Glass powder, initiator, silica, substituted pyrimidine, calcium hydroxide, peroxy compound, pigment	Methacrylated phosphoric ester, dimethacrylate, acetate, stabilizer, initiator	Resin cement, self-etching	3 M ESPE (Seefeld, Germany)	In capsules	Yes
Ketac Cem Aplicap	Glass powder, pigments	Polycarboxylic acid, tartaric acid, water, conservation agents	Glass ionomer, acid-base reacting	3 M ESPE (Seefeld, Germany)	In capsules	Yes
Harvard zinc phosphate cement	Zinc oxide, magnesium oxide	o-phosphoric acid	Zinc phosphate, acid-base reacting	Harvard Dental International GmbH (Hoppegarten, Germany)	3/2	No
Ceramir Crown & Bridge	Calcium aluminate, strontium fluoride, polyacrylic acid, tartaric acid, strontium aluminofluoride glass	Water, accelerators	Bioceramic, acid-base reacting	Doxa AB (Uppsala, Sweden)	3.2/1	Yes
Calcium aluminate cement reference material	Calcium aluminate	Water, accelerators	Bioceramic, acid-base reacting	Doxa AB (Uppsala, Sweden)	2.5/1	No
Glass ionomer cement reference material	Poly acrylic acid, tartaric acid, strontium aluminofluoride glass, strontium fluoride	Water	Glass ionomer, acid-base reacting	Doxa AB (Uppsala, Sweden)	3.2/1	Yes

**Table 2:** Contents of the reference, control, and test solutions for measuring bactericidal effects of varying pH.

Contents (mL)				
Solution	BHI broth	pH buffer	H_2_O	*S. mutans *(OD_600_ = 1.0)
Reference	4	—	6	—
Control	3.4	—	6	0.6
Test solutions	3.4	6	—	0.6
